# Enhancement of Countermovement Jump Performance Using a Heavy Load with Velocity-Loss Repetition Control in Female Volleyball Players

**DOI:** 10.3390/ijerph182111530

**Published:** 2021-11-02

**Authors:** Michal Krzysztofik, Rafal Kalinowski, Robert Trybulski, Aleksandra Filip-Stachnik, Petr Stastny

**Affiliations:** 1 Institute of Sport Sciences, The Jerzy Kukuczka Academy of Physical Education in Katowice, 40-065 Katowice, Poland; a.filip@awf.katowice.pl; 2 Department of Exercise and Sport Performance, The Jerzy Kukuczka Academy of Physical Education in Katowice, 40-065 Katowice, Poland; r.kalinowski@awf.katowice.pl; 3 Department of Medical Sciences, The Wojciech Korfanty School of Economics, 40-659 Katowice, Poland; rtrybulski@o2.pl; 4 Provita Zory Medical Center, 44-240 Zory, Poland; 5 Department of Sport Games, Faculty of Physical Education and Sport, Charles University, 16252 Prague, Czech Republic; stastny@ftvs.cuni.cz

**Keywords:** movement velocity, sport science, post activation potentiation, resistance training, power

## Abstract

Although velocity control in resistance training is widely studied, its utilization in eliciting post-activation performance enhancement (PAPE) responses receives little attention. Therefore, this study aimed to evaluate the effectiveness of heavy-loaded barbell squats (BS) with velocity loss control conditioning activity (CA) on PAPE in subsequent countermovement jump (CMJ) performance. Sixteen resistance-trained female volleyball players participated in this study (age: 24 ± 5 yrs.; body mass: 63.5 ± 5.2 kg; height: 170 ± 6 cm; relative BS one-repetition maximum (1RM): 1.45 ± 0.19 kg/body mass). Each participant performed two different conditions: a set of the BS at 80% 1 RM with repetitions performed until a mean velocity loss of 10% as the CA or a control condition without CA (CNTRL). To assess changes in jump height (JH) and relative mean power output (MP), the CMJ was performed 5 min before and throughout the 10 min after the CA. The two-way analysis of variance with repeated measures showed a significant main effect of condition (*p* = 0.008; η^2^ = 0.387) and time (*p* < 0.0001; η^2^ = 0.257) for JH. The post hoc test showed a significant decrease in the 10th min in comparison to the value from baseline (*p* < 0.006) for the CNTRL condition. For the MP, a significant interaction (*p* = 0.045; η^2^ = 0.138) was found. The post hoc test showed a significant decrease in the 10th min in comparison to the values from baseline (*p* < 0.006) for the CNTRL condition. No significant differences were found between all of the time points and the baseline value for the CA condition. The CA used in the current study fails to enhance subsequent countermovement jump performance in female volleyball players. However, the individual analysis showed that 9 out of the 16 participants (56%) responded positively to the applied CA, suggesting that the PAPE effect may be individually dependent and should be carefully verified before implementation in a training program.

## 1. Introduction

Post-activation performance enhancement (PAPE) is a short-term improvement in explosive performance due to prior muscle activation. The occurrence of performance enhancement may be related to mechanisms such as an increase in muscle temperature, fiber water content, and muscle activation [1]. In training practice, the improvement effect is obtained by applying a conditioning activity (CA) prior to an explosive movement with a similar movement pattern [2]. In recent years, many studies have been undertaken to determine the optimal parameters of CA that are conducive to reaching peak performance. This concerned the determination of the optimal type of exercise used for CA [3,4], optimal intensity and volume [5,6,7,8], as well as rest intervals [9]. Investigations indicated that PAPE was obtained over a wide range of intensities (from plyometric body-weight to supramaximal loads) and that high loads are the most studied (~80–90% one-repetition maximum [1RM]) [2,10,11,12,13]. However, there is a lack of specific guidelines for the optimal volume of a CA. Besides, findings point out that not only CA variables but also the individual characteristics of the participants, such as the type of muscle fibers, training experience, muscle strength, and fatigue resistance, are also of key importance [2,10].

There is a consensus that one of the key determinants to induce PAPE is the balance between potentiation elicited by the CA and fatigue, which co-exist and affect performance outcomes [14]. Muscle performance may be enhanced if potentiation dominates over fatigue, or remain unchanged if fatigue and potentiation are at similar levels, and decrease if fatigue dominates over potentiation [2]. Therefore, the recently proposed method of controlling velocity loss during CA, which takes into account the athlete’s fatigue profile, seems to be a promising solution [15,16]. Velocity-based resistance training is used as a practical method for monitoring and quantifying the intensity and volume of resistance exercise. Recording velocity loss during successive repetitions may serve as a sensitive indicator of neuromuscular fatigue [17]. Velocity loss control can also be useful during CA to maintain the optimal balance between potentiation and fatigue, and consequently contribute to PAPE induction. However, to date, only three studies have investigated the use of velocity loss control during a CA to optimize PAPE [15,16,18]. Tsoukos et al. [15,16] showed that when velocity control (mean velocity could not drop by more than 10% of the mean velocity from the first repetition) was used during CA (bench press with 40%, 60%, and 80% 1 RM), the mean propulsive velocity during the post-activation exercise (bench press throw at 30% 1 RM) significantly increased by 4.5–8% among resistance-trained males. Further, both studies confirmed that greater PAPE enhancement was reached after a 10% than a 30% movement velocity loss, which confirms that velocity loss control can be useful to the optimization of CA. Additionally, these studies also showed that during velocity loss CA, heavier loads (80% 1 RM) may be slightly more effective than lighter loads (80% and 60% vs. 40% 1 RM; effect size: 0.7–0.8 vs. 0.4; 7–9% vs. 6%, respectively). However, it is also worth noting that in both studies there were considerable differences in the number of performed repetitions during CA to a 10% movement velocity-loss between participants (from two to five repetitions), which justifies an individual approach to the induction of PAPE. Moreover, such a CA protocol has not yet been replicated for PAPE in the lower limbs or on female athletes. According to acknowledged studies, males present higher motor unit firing frequency [19,20] along with a greater cross-sectional area of type II fibers [19,21] and relatively lower oxidative capacity [20], which could lead to a faster rise in fatigue levels compared to females. Furthermore, the neuromuscular responses of females appear to be delayed, [22,23] which, along with a lower level of muscular strength compared to males, may affect PAPE response. Based on the above, the effect of a CA on subsequent performance may develop in different ways in males and females. Although Wilson et al. [10] did not report significant differences between the genders, a slightly greater degree of PAPE was found among males than females (effect size: 0.42 vs. 0.2). Furthermore, Rixon et al. [24], found a smaller PAPE effect on countermovement jump among females in comparison to males, after a voluntary maximal isometric squat. Therefore, there is a reason to believe that the magnitude of the PAPE effect may be sex-dependent. In addition, while the effectiveness of heavy loads with velocity-loss control CA in eliciting the PAPE effect was investigated in the upper body exercise [15,16,18], as far as we know, for lower body exercise it is unknown. Therefore, the determination of the effectiveness of this type of CA to improve lower body performance in female athletes provides new perspectives that shed light on the use of velocity-controlled CA in a broader range of athletic populations.

Since velocity loss control in resistance training allows the same level of fatigue to be induced, irrespective of the individual characteristics of the participant, it may be useful in optimizing the CA in the attempt to elicit a PAPE effect. However, to date, no studies have been conducted that use the velocity loss CA protocol in optimizing the PAPE for the lower body among females. Therefore, the aim of this study was to evaluate the optimal rest interval of heavy-load barbell squats with velocity loss CA on PAPE in subsequent countermovement jump performance. We hypothesized that according to the research carried out so far, a 10% decrease in mean velocity during CA significantly increases subsequent countermovement jump performance.

## 2. Materials and Methods

### 2.1. Experimental Approach to the Problem

**Figure 1:**
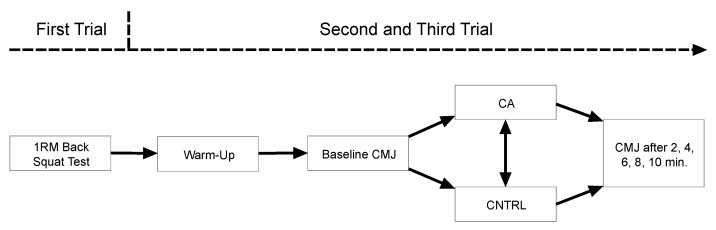
Study design. 1RM—one repetition maximum; CMJ—countermovement jump; CA—condition used velocity controlled conditioning activity; CNTRL—control condition.

The participants took part in a familiarization session and two experimental sessions within 3 weeks. The familiarization session included the determination of the 1 RM load of the back squat and the performance of two sets until 10% velocity loss at 80%1RM. The experimental sessions were performed in randomized order, one week apart, and each participant performed a single set of back squats at 80% 1 RM until mean movement velocity dropped by 10% as the CA (CA) or a control condition, in which the participants did not perform any CA (CNTRL) (Figure 1). To assess changes in jump height (JH) and mean power output (MP), single sets of two repetitions of the countermovement jump (CMJ) were performed before and after the CA in five time points with 2 min rest intervals.

### 2.2. Subjects

Sixteen resistance-trained amateur female volleyball players (age: 24 ± 5 yrs.; body mass: 63.5 ± 5.2 kg; height: 170 ± 6 cm; body fat: 19.5 ± 4.6%; experience in resistance training: 3 ± 4; experience in volleyball training: 13 ± 4 yrs.; relative back squat one-repetition maximum [1RM]: 1.45 ± 0.19 kg/body mass) participated in this study. The inclusion criteria were as follows: (i) free from neuromuscular and musculoskeletal disorders, (ii) at least two years’ experience in resistance training and heavy-loaded back squats, (iii) able to perform a back squat with a load of at least 120% of their body mass, (iv) self-described satisfactory health status. Participants were excluded if they reported: (i) irregular participation in resistance training less than two times a week for the last six months, (ii) regular caffeine supplementation. Moreover, if a participant obtained an increase in CMJ height at any time point after the CA compared with the baseline measurement, she was considered as a positive; if not, then as a negative respondent. The participants were instructed to not perform any additional resistance exercises within 72 h of testing to avoid fatigue. Moreover, they were asked to maintain their normal dietary and sleep habits throughout the study and not to use any supplements or stimulants for 24 h prior to the sessions. The study participants were allowed to withdraw from the experiment at any moment. They were informed about the benefits and potential risks of the study before providing their written informed consent for participation. The study protocol was approved by the Bioethics Committee for Scientific Research, at the Academy of Physical Education in Katowice, Poland (3/2021), and performed according to the ethical standards of the Declaration of Helsinki, 2013. To calculate the sample size, statistical software (G*Power, Dusseldorf, Germany) was used. Given the two-way analysis of variance (ANOVA) (two conditions and six repeated measures), the small overall effect size (ES) = 0.31, an alpha-error < 0.05, the desired power (1-ß error) = 0.8, and the correlation between the repeated measures = 0.85, the total sample size resulted in five participants. This value of effect size was chosen according to findings from Seitz and Haff [2] on the impact of PAPE on jumping performance.

### 2.3. Procedures

#### 2.3.1. Familiarization Session and 1 RM Strength Test

The participants arrived in the laboratory at the same time of day as the upcoming experimental sessions (in the evening between 5:00 and 7:00 pm). A week before the main experiment, the 1 RM back squat test was performed according to the recommendations proposed by Gepfert et al. [25]. The participants performed a standardized warm-up consisting of cycling on a stationary bike for 5 min (Keiser M3 Indoor Bike, Keiser Corporation, Fresno, CA, USA) at a resistance of approximately 100 W and a cadence within 70–80 rpm; 2 circuits of 10 trunk rotations and side-bends; 10 internal, external and lateral arm swings; 10 bodyweight squats; and 10 push-ups. Next, the participants performed 10, 6, 4, and 3 repetitions respectively, starting at a load of 20 kg and progressing to 60–80% of their estimated 1RM. The first testing load was set to an estimated 80% 1 RM and was increased by 2.5–5 kg for each subsequent attempt until the participant was unable to perform a lift with the proper technique. The participants were instructed to perform each repetition with an eccentric phase of 2 s duration and a maximal velocity in the concentric phase of the movement [26,27]. The participants started from an upright position, with the knees and hips fully extended, their stance approximately shoulder-width apart, with both feet positioned flat on the floor in parallel or externally rotated to a maximum of 15° [28]. The stance width and foot position were individually adjusted and carefully replicated on every lift. From this position, the participants were required to descend until making contact with the bench and then to perform the concentric phase of the movement in an explosive manner [29,30]. The height of the bench was individually selected and allowed each participant to descend with the hips below the knee line, according to the rules of the International Powerlifting Federation [31]. The 1 RM was defined as the highest load completed without any help from the spotters. Five-minute rest intervals were allowed between the 1 RM attempts, and all the 1 RM values were obtained within five attempts.

Following the 1 RM test, all the participants performed two sets of back squats until a 10% mean velocity loss at 80% 1 RM.

#### 2.3.2. Experimental Sessions

In a randomized and counterbalanced order, after performing an identical warm-up to the one performed before the 1 RM test, the participants performed two different testing conditions, one-week apart: a single set of back squats at 80% 1 RM, with repetitions performed until mean movement velocity dropped by 10% as the CA (CA), or a control condition, in which the participants did not perform CA (CNTRL). To assess changes in JH and MP, single sets of two repetitions of the countermovement jump (CMJ) were performed. The CMJ was performed 5 min before and 2, 4, 6, 8, 10 min after the CA. The best repetition was kept for further analysis.

#### 2.3.3. Measurement of Movement Velocity during the Conditioning Activity

To assess the mean velocity loss during the CA, a GymAware Powertool (Kinetic Performance Technology, Canberra, Australia) linear position transducer was used. This device provides reliable and valid data [32]. The external end of the cable was attached to the side of the bar and provided no resistance. The device was placed on the floor directly under the bar, with the magnetic bottom positioned on a weight plate to ensure no movement during each lift. The velocity of the barbell was recorded at 50 Hz.

#### 2.3.4. Measurement of Countermovement Jump Performance with Arm Swing

The CMJ starting position was a standing position with a straight torso and the knees fully extended, with the feet shoulder-width apart and the hands free to move. This type of jump was chosen because it more closely replicates the competitive conditions in volleyball. The participants were instructed to perform a quick downward movement (approximately 90° of knee flexion), and afterwards a fast upward movement to jump as high as possible. The Optojump photoelectric cells (Microgate, Bolzano, Italy) device is an infrared platform with proven validity and reliability for assessing vertical jump height. The device measures the flight of vertical jumps with a sampling frequency of 1000 Hz. The intra-class correlation coefficient and coefficient of variation were 0.97 and 5.1% for JH, while for MP they were 0.97 and 7.9%, respectively.

## 3. Statistical Analyses

All the statistical analyses were performed using SPSS (version 25.0; SPSS, Inc., Chicago, IL, USA) and were expressed as means with standard deviations (±SD). Moreover, the 95% confidence intervals for the mean values and relative differences (i.e., in percentages) between the baseline and post-CA values were also calculated. Statistical significance was set at *p* < 0.05. The normality of the data distribution was checked using Shapiro–Wilk tests. A logarithm transformation was used on JH to obtain a normal distribution. Next, the acute effects of the CA on the dependent variables were examined by two-way repeated-measures ANOVA (2 conditions × 6 time points). The effect size was determined by partial eta squared (η^2^). The partial eta squared values were classified as small (0.01 to 0.059), moderate (0.06 to 0.137), and large (>0.137). Furthermore, the differences in the relative 1 RM back squat, the repetitions performed during the CA, resistance training as well as volleyball training experience between participants who positively and negatively responded to CA were examined by an independent samples *t*-test. The magnitude of mean differences was expressed with standardized effect sizes; the thresholds for the qualitative descriptors of Cohen’s d were defined: <0.20 as “trivial”, 0.20–0.49 as “small”, 0.50–0.79 as “moderate”, and >0.80 as “large” [33].

## 4. Results

The individual analysis showed that 9 out of the 16 participants (56%) responded positively to the applied CA. The independent samples *t*-test indicated a non-significant difference in relative 1 RM back squat (1.5 ± 0.2 vs. 1.38 ± 0.17 kg/bm), repetitions performed during the CA (4 ± 1.2 vs. 4.1 ± 1.5) (Table 1), resistance training (4.1 ± 2.4 vs. 2.6 ± 0.5 yrs.), as well as volleyball training experience (14 ± 4.8 vs. 10.9 ± 3.1 yr) between participants who positively and negatively responded to the CA (*p* = 0.246, *p* = 0.835, *p* = 0.116, *p* = 0.16; respectively). The Shapiro–Wilk tests indicated that the normality of the data was violated for JH; therefore, a logarithm transformation was used to obtain a normal distribution.

### 4.1. Jump Height

The two-way ANOVA (2 conditions × 6 rest time points) showed a significant main effect of condition (*p* = 0.008; η^2^ = 0.387) and time (*p* < 0.0001; η^2^ = 0.257), but not interaction (*p* = 0.054; η^2^ = 0.133) for JH. The post hoc test showed a significant decrease in the 10th min in comparison to the value from baseline (*p* < 0.006) for the CNTRL condition. Moreover, JH during the CNTRL was significantly higher in comparison with the corresponding values during the CA condition from the 4th to the 8th min of the rest-time interval (*p* = 0.024; *p* = 0.021; *p* = 0.013) (Table 2). The evaluation of participants who positively responded to CA (*n* = 9) showed an increase in JH from baseline to the best post-CA countermovement jump performance of 2.8 ± 4.9% (from 37.4 ± 5.4 cm to 38.7 ± 7.1 cm; ES: 0.21) during the CNTRL and of 6.1 ± 4.8% (from 36.1 ± 4.5 cm to 38.4 ± 6.1 cm; ES: 0.43) during the CA condition.

### 4.2. Mean Power Output

The two-way ANOVA (2 conditions × 6 rest time points) showed a significant interaction (*p* = 0.045; η^2^ = 0.138) for the MP. The post hoc test showed a significant decrease in the 10th min in comparison to the values from baseline (*p* < 0.006) for the CNTRL condition (Table 2). No significant differences were found between all of the time points and the baseline value for the CA condition. Moreover, the MP during the CNTRL was significantly higher in comparison with the corresponding values during the CA condition from the 4th to the 8th min of the rest-time interval (*p*= 0.001; *p* = 0.009; *p* = 0.006). The evaluation of the participants who positively responded to the CA (*n* = 9) showed an increase in the MP from baseline to the best post-CA countermovement jump performance of 3.7 ± 7.1% (from 19.56 ± 3.68 W/kg to 20.45 ± 4.77 W/kg; ES: 0.21) during the CNTRL and of 8.4 ± 6.3% (from 18.6 ± 3.06 W/kg to 20.25 ± 4.09 W/kg; ES: 0.46) during the CA condition.

## 5. Discussion

This study aimed to evaluate the effects of a single set of heavy-loaded barbell squats with velocity loss on PAPE in subsequent countermovement jump performance. The main finding of this study was that the CA used fails to enhance the subsequent countermovement jump performance in female volleyball players. However, the individual analysis showed that 9 out of the 16 participants (56%) responded positively to the applied CA (by 6.1 ± 4.8% in JH and 8.4 ± 6.3% in MP), suggesting that the PAPE effect may be individually dependent. Therefore, the CA used might be insufficient to enhance subsequent performance in some cases; hence, it should be individually verified before possible implementation in training programs.

The results of the present study are in agreement with previous research, which found PAPE response to be highly individualized [34,35,36]. The balance between fatigue and potentiation has been suggested as the main factor affecting the amount of PAPE expressed after CA [14]. Hence, the key to triggering PAPE may be a personalized approach to adjusting the training variables of the CA (volume and intensity). Stronger and more experienced individuals may develop fatigue resistance under higher loads [34,37]. Taking this into account, it can be postulated that stronger and weaker individuals may react differently to various components of the CA. However, it seems that relative strength and training experience were not major factors in the current study. The average level of the relative 1 RM back squat strength of the participants in the present study was 1.45 ± 0.19, which means that they were classified as low strength level, according to Seitz and Haff [2]. They indicated that weaker (≤1.5) and stronger (>1.5 relative back squat strength) individuals seem to exhibit different PAPE responses, and the larger magnitude of the PAPE effect is observed among stronger individuals and those with more experience in resistance training. However, the level of relative strength, as well as experience in resistance training, of the positive- and negative-response participants did not differ significantly (1.5 ± 0.2 vs. 1.38 ± 0.17 kg/bm; 4.1 ± 2.4 vs. 2.6 ± 0.5 yr; respectively), nor with participants from studies that demonstrated performance enhancement after velocity-controlled CA [15,16] (at least 3 years). Therefore, it seems that the obtained results could have been influenced by other factors.

Although previous studies have shown that velocity-controlled CA is an effective method for optimizing PAPE [15,16,18], this was not confirmed for the group in the present study as a whole. Tsoukos et al. [15,16] showed that bench press throw performance was significantly improved after CA (bench press; external loads 40; 60 or 80% 1RM) when the velocity dropped by 10 and 30% of the first repetition among physically active males. Furthermore, the authors also found that a greater level of explosive performance improvement was achieved by applying a lower velocity loss threshold and a higher load compared to a higher velocity loss and lower load (80%1RM with 10% velocity-loss vs. 40% and 60% 1 RM with 10% and 30% velocity loss). However, the present study did not show such a positive effect for the cohort as a whole after a velocity-controlled CA at 80% 1 RM with a 10% velocity-loss. Unfortunately, to our knowledge, velocity-controlled CA was used in male groups and involved upper-body exercises; hence, any comparison with our results should be approached with caution. The failure to improve CMJ performance after the CA used in our study may be related to the engaged part of the body and/or gender of the participants. In the present study, the volume of the CA was individualized for each participant, so it seems that the degree of neuromuscular fatigue as a result of the velocity loss assessment was similar. The participants performed only a single set of the CA (approximately ~4.1 ± 1.3 repetitions), which might be insufficient to target the mechanisms underpinning PAPE, due the increase in muscle activation and temperature and/or intramuscular fluid accumulation [1]. In addition, lower-limb musculature exceeds that of the upper limbs; thus, a higher volume of CA may be necessary to elicit the PAPE effect. As indicated by Seitz and Haff [2], a multiple-set CA especially is more effective at inducing the PAPE effect in weaker individuals, as in the current study. Considering the above, the fact that female neuromuscular responses appear to be delayed along, with their superior fatigue resistance to males [20,23], and, finally that a slightly lower degree of PAPE was found among females in comparison to males [10,24], perhaps a multiple-set CA would have been more effective for the participants in this study. Nevertheless, it should be noted that out of all the participants in the experiment, in nine of them, CMJ performance increased after velocity-controlled CA (by 6.1 ± 4.8% in JH), while in seven, the performance decreased (by −2.9 ± 2.7% in JH). Therefore, such a variety of responses among the participants confirms that the individual characteristics of the participant can be a determining factor in inducing the PAPE effect.

Further, the post-CA measurement was performed in the range of 2 to 10 min; therefore, in certain cases, a longer rest-time interval may be required to induce the PAPE effect. On the other hand, the influence of individual time points on consecutive measurements cannot be ruled out either. Similar protocols for assessing the PAPE effect have been used previously in the other studies [4,15,16]; however, we cannot be sure that this did not affect the performance due to the raising fatigue level. Therefore, there is a particular need to establish the PAPE response in a wide range of rest-time intervals for each participant before implementing the PAPE protocol in a training regimen. Moreover, further investigations to assess the influence of multiple-set velocity-controlled CA on subsequent performance and compare them with other conditions are required (with a high-loaded fixed volume and plyometric CA) especially among female and mixed-sex groups.

In addition to those already mentioned, we are aware that our research may have other limitations. First, we analyzed only a single CA protocol’s effectiveness at inducing the PAPE effect. Therefore, future studies using different CA exercises with variations in intensity (with lower or higher loads), velocity thresholds (especially with greater velocity decreases), and rest intervals could help to optimize PAPE responses. It also seems necessary to assess the repeatability of the rest- intervals in successive workouts as well as after long periods of training. Second, we enrolled amateur female volleyball athletes with low relative strength levels; thus, the results of this study may not translate to alternative samples. Such studies could provide additional information on the protocols for eliciting PAPE, which may require differentiation between athletes, and on whether the obtained performance improvement reaches a similar magnitude. Lastly, no physiological variables, nor any biomechanical or electromyographical analysis, were assessed that could help to explain the obtained results, such as the reasons for individual responses to PAPE.

In conclusion, performance changes varied between the participants under CA and CNTRL conditions, with performance changes ranging from −8% to 15% compared with the pre-CA values. Nevertheless, it seems that other individual factors alongside relative strength and training experience may influence the response to a velocity-controlled CA. Gullich and Schmidtbleicher [35] concluded that PAPE response varied greatly between individuals, which was apparent in the current study. Perhaps the explanation of these results lies in genetics and requires direct analysis, or at least an indirect prediction of the composition of muscle fibers using multiple-repetition tests [36]. Therefore, to optimize PAPE, both CA velocity control and the evaluation of its effectiveness in post-activation exercise individual for each participant are necessary.

## 6. Conclusions

The current study provides novel insights into the monitoring and prescription of CA to induce PAPE response. Based on the main findings of this study, it can be concluded that a CA consisting of a single set of 80% 1 RM back squat to a velocity loss of 10% fails to enhance subsequent CMJ performance among amateur volleyball female athletes. However, 9 out of the 16 participants improved their CMJ performance after the CA, which indicates that the PAPE effect may be individually dependent. In this regard, coaches and athletes are advised to establish whether the CA sufficiently enhances performance before recommending or rejecting PAPE protocols.

## Figures and Tables

**Table 1:** Characteristics of the conditioning activity (back squat using 80% of one-repetition maximum) in the 10% mean velocity-loss trial.

Variable	Mean ± SD
Mean Lifted Load [kg]	71.3 ± 8.5
Mean Number of Repetitions [n]	4.1 ± 1.3
Maximal Number of Repetitions [n]	6
Minimal Number of Repetitions [n]	2

**Table 2:** Comparison of countermovement jump performance between two conditions.

	Baseline (95CI)	2 min Rest (95CI)	4 min Rest (95CI)	6 min Rest (95CI)	8 min Rest (95CI)	10 min Rest (95CI)
Jump Height [cm]						
CNTRL	37.8 ± 5.8 (34.7 to 40.9)	37.9 ± 6.2 (34.5 to 41.2)	38.6 ± 7.1 (34.8 to 42.4)	38.2 ± 7.2 (34.4 to 42)	36.7 ± 7 (32.9 to 40.4)	36.3 ± 6.3 * (32.9 to 39.6)
CA	37.1 ± 4.8 (34.5 to 39.6)	36.6 ± 6.4 (33.2 to 40)	36.5 ± 6 ^#^ (33.3 to 39.7)	36.3 ± 6.3 ^#^ (33 to 39.6)	35.6 ± 6.5 ^#^ (32.1 to 39)	36.2 ± 6.1 (33 to 39.4)
Relative Mean Power [W/kg]						
CNTRL	20.54 ± 4.29 (18.25 to 22.82)	20.59 ± 4.57 (18.16 to 23.03)	21.11 ± 5.07 (18.41 to 23.81)	20.82 ± 5.13 (18.09 to 23.56)	19.72 ± 5.15 (16.97 to 22.46)	19.45 ± 4.76 * (16.92 to 21.99)
CA	20.01 ± 3.7 (18.04 to 21.98)	19.66 ± 4.63 (17.19 to 22.13)	19.59 ± 4.41 ^#^ (17.24 to 21.94)	19.48 ± 4.54 ^#^ (17.06 to 21.9)	18.96 ± 4.84 ^#^ (16.38 to 21.54)	19.36 ± 4.42 (17.01 to 21.72)

Results are mean ± SD (95% confidence intervals); CA—condition used velocity controlled conditioning activity; CNTRL—control condition; * significant difference between baseline values (*p* < 0.05); ^#^ significant difference between conditions in comparison with the corresponding time-point (*p* < 0.05).

## Data Availability

The datasets analyzed during the current study are available from the corresponding author on reasonable request.

## References

[1] Blazevich A.J., Babault N. (2019). Post-Activation Potentiation versus Post-Activation Performance Enhancement in Humans: Historical Perspective, Underlying Mechanisms, and Current Issues. Front. Physiol..

[2] Seitz L.B., Haff G.G. (2016). Factors Modulating Post-Activation Potentiation of Jump, Sprint, Throw, and Upper-Body Ballistic Performances: A Systematic Review with Meta-Analysis. Sports Med..

[3] Esformes J.I., Keenan M., Moody J., Bampouras T.M. (2011). Effect of Different Types of Conditioning Contraction on Upper Body Postactivation Potentiation. J. Strength Cond. Res..

[4] Bogdanis G.C., Tsoukos A., Veligekas P., Tsolakis C., Terzis G. (2014). Effects of Muscle Action Type with Equal Impulse of Conditioning Activity on Postactivation Potentiation. J. Strength Cond. Res..

[5] Krzysztofik M., Wilk M., Golas A., Lockie R.G., Maszczyk A., Zajac A. (2020). Does Eccentric-Only and Concentric-Only Activation Increase Power Output?. Med. Sci. Sports Exerc..

[6] Krzysztofik M., Wilk M., Lockie R.G., Golas A., Zajac A., Bogdanis G.C. (2020). Postactivation Performance Enhancement of Concentric Bench Press Throw after Eccentric-Only Conditioning Exercise. J. Strength Cond. Res..

[7] Matusiński A., Pietraszewski P., Krzysztofik M., Gołaś A. (2021). The Effects of Resisted Post-Activation Sprint Performance Enhancement in Elite Female Sprinters. Front. Physiol..

[8] Khamoui A.V., Brown L.E., Coburn J.W., Judelson D.A., Uribe B.P., Nguyen D., Tran T., Eurich A.D., Noffal G.J. (2009). Effect of Potentiating Exercise Volume on Vertical Jump Parameters in Recreationally Trained Men. J. Strength Cond. Res..

[9] Kilduff L.P., Bevan H.R., Kingsley M.I.C., Owen N.J., Bennett M.A., Bunce P.J., Hore A.M., Maw J.R., Cunningham D.J. (2007). Postactivation Potentiation in Professional Rugby Players: Optimal Recovery. J. Strength Cond. Res..

[10] Wilson J.M., Duncan N.M., Marin P.J., Brown L.E., Loenneke J.P., Wilson S.M.C., Jo E., Lowery R.P., Ugrinowitsch C. (2013). Meta-Analysis of Postactivation Potentiation and Power: Effects of Conditioning Activity, Volume, Gender, Rest Periods, and Training Status. J. Strength Cond. Res..

[11] Krzysztofik M., Wilk M., Filip A., Zmijewski P., Zajac A., Tufano J.J. (2020). Can Post-Activation Performance Enhancement (PAPE) Improve Resistance Training Volume during the Bench Press Exercise?. Int. J. Environ. Res. Public Health.

[12] Krzysztofik M., Wilk M., Stastny P., Golas A. (2021). Post-Activation Performance Enhancement in the Bench Press Throw: A Systematic Review and Meta-Analysis. Front. Physiol..

[13] Krzysztofik M., Wilk M. (2020). The Effects of Plyometric Conditioning on Post-Activation Bench Press Performance. J. Hum. Kinet..

[14] Rassier D.E., Macintosh B.R. (2000). Coexistence of Potentiation and Fatigue in Skeletal Muscle. Braz. J. Med Biol. Res..

[15] Tsoukos A., Brown L.E., Veligekas P., Terzis G., Bogdanis G.C. (2019). Postactivation Potentiation of Bench Press Throw Performance Using Velocity-Based Conditioning Protocols with Low and Moderate Loads. J. Hum. Kinet..

[16] Tsoukos A., Brown L.E., Terzis G., Veligekas P., Bogdanis G.C. (2021). Potentiation of Bench Press Throw Performance Using a Heavy Load and Velocity-Based Repetition Control. J. Strength Cond. Res..

[17] Rodríguez-Rosell D., Yáñez-García J.M., Mora-Custodio R., Pareja-Blanco F., Ravelo-García A.G., Ribas-Serna J., González-Badillo J.J. (2020). Velocity-Based Resistance Training: Impact of Velocity Loss in the Set on Neuromuscular Performance and Hormonal Response. Appl. Physiol. Nutr. Metab..

[18] Krzysztofik M., Matykiewicz P., Celebanska D., Jarosz J., Gawel E., Zwierzchowska A. (2021). The Acute Post-Activation Performance Enhancement of the Bench Press Throw in Disabled Sitting Volleyball Athletes. Int. J. Environ. Res. Public Health.

[19] Pincivero D.M., Coelho A.J., Campy R.M. (2003). Perceived Exertion and Maximal Quadriceps Femoris Muscle Strength during Dynamic Knee Extension Exercise in Young Adult Males and Females. Eur. J. Appl. Physiol..

[20] Pincivero D.M., Gandaio C.B., Ito Y. (2003). Gender-Specific Knee Extensor Torque, Flexor Torque, and Muscle Fatigue Responses during Maximal Effort Contractions. Eur. J. Appl. Physiol..

[21] Mihic S., MacDONALD J.R., McKENZIE S., Tarnopolsky M.A. (2000). Acute Creatine Loading Increases Fat-Free Mass, but Does Not Affect Blood Pressure, Plasma Creatinine, or CK Activity in Men and Women. Med. Sci. Sports Exerc..

[22] Marotta N., Demeco A., de Scorpio G., Indino A., Iona T., Ammendolia A. (2020). Late Activation of the Vastus Medialis in Determining the Risk of Anterior Cruciate Ligament Injury in Soccer Players. J. Sport Rehabil..

[23] Marotta N., Demecon A., Moggio L., Isabello L., Iona T., Ammendolia A. (2020). Correlation between Dynamic Knee Valgus and Quadriceps Activation Time in Female Athletes. J. Phys. Educ. Sport.

[24] Rixon K.P., Lamont H.S., Bemben M.G. (2007). Influence of Type of Muscle Contraction, Gender, and Lifting Experience on Postactivation Potentiation Performance. J. Strength Cond. Res..

[25] Gepfert M., Krzysztofik M., Kostrzewa M., Jarosz J., Trybulski R., Zajac A., Wilk M. (2020). The Acute Impact of External Compression on Back Squat Performance in Competitive Athletes. Int. J. Environ. Res. Public Health.

[26] Wilk M., Golas A., Zmijewski P., Krzysztofik M., Filip A., Coso J.D., Tufano J.J. (2020). The Effects of the Movement Tempo on the One-Repetition Maximum Bench Press Results. J. Hum. Kinet..

[27] Wilk M., Gepfert M., Krzysztofik M., Mostowik A., Filip A., Hajduk G., Zajac A. (2020). Impact of Duration of Eccentric Movement in the One-Repetition Maximum Test Result in the Bench Press among Women. J. Sports Sci. Med..

[28] Martínez-Cava A., Morán-Navarro R., Sánchez-Medina L., González-Badillo J.J., Pallarés J.G. (2019). Velocity- and Power-Load Relationships in the Half, Parallel and Full Back Squat. J. Sports Sci..

[29] Pallarés J.G., Sánchez-Medina L., Pérez C.E., De La Cruz-Sánchez E., Mora-Rodriguez R. (2014). Imposing a Pause between the Eccentric and Concentric Phases Increases the Reliability of Isoinertial Strength Assessments. J. Sports Sci..

[30] Sánchez-Medina L., Pallarés J., Pérez C., Morán-Navarro R., González-Badillo J. (2017). Estimation of Relative Load From Bar Velocity in the Full Back Squat Exercise. Sports Med. Int. Open.

[31] Wilk M., Krzysztofik M., Bialas M. (2020). The Influence of Compressive Gear on Maximal Load Lifted in Competitive Powerlifting. Biol. Sport.

[32] Banyard H.G., Nosaka K., Sato K., Haff G.G. (2017). Validity of Various Methods for Determining Velocity, Force, and Power in the Back Squat. Int. J. Sports Physiol. Perform..

[33] Cohen J. (2013). Statistical Power Analysis for the Behavioral Sciences.

[34] Chiu L.Z.F., Fry A.C., Weiss L.W., Schilling B.K., Brown L.E., Smith S.L. (2003). Postactivation Potentiation Response in Athletic and Recreationally Trained Individuals. J. Strength Cond. Res..

[35] Evetovich T.K., Conley D.S., McCawley P.F. (2015). Postactivation Potentiation Enhances Upper- and Lower-Body Athletic Performance in Collegiate Male and Female Athletes. J. Strength Cond. Res..

[36] McCann M.R., Flanagan S.P. (2010). The Effects of Exercise Selection and Rest Interval on Postactivation Potentiation of Vertical Jump Performance. J. Strength Cond. Res..

[37] Hamada T., Sale D.G., MacDougall J.D., Tarnopolsky M.A. (2000). Postactivation Potentiation, Fiber Type, and Twitch Contraction Time in Human Knee Extensor Muscles. J. Appl. Physiol..

